# Terahertz Emitter Using Resonant-Tunneling Diode and Applications

**DOI:** 10.3390/s21041384

**Published:** 2021-02-16

**Authors:** Masahiro Asada, Safumi Suzuki

**Affiliations:** 1 Institute of Innovative Research, Tokyo Institute of Technology, Tokyo 152-8552, Japan; 2 Department of Electrical and Electronic Engineering, Tokyo Institute of Technology, Tokyo 152-8552, Japan; suzuki.s.av@m.titech.ac.jp

**Keywords:** terahertz oscillator, resonant-tunneling diode, frequency tuning, spectral narrowing, polarizations, spectroscopy, wireless communication, radar

## Abstract

A compact source is important for various applications utilizing terahertz (THz) waves. In this paper, the recent progress in resonant-tunneling diode (RTD) THz oscillators, which are compact semiconductor THz sources, is reviewed, including principles and characteristics of oscillation, studies addressing high-frequency and high output power, a structure which can easily be fabricated, frequency tuning, spectral narrowing, different polarizations, and select applications. At present, fundamental oscillation up to 1.98 THz and output power of 0.7 mW at 1 THz by a large-scale array have been reported. For high-frequency and high output power, structures integrated with cylindrical and rectangular cavities have been proposed. Using oscillators integrated with varactor diodes and their arrays, wide electrical tuning of 400–900 GHz has been demonstrated. For spectral narrowing, a line width as narrow as 1 Hz has been obtained, through use of a phase-locked loop system with a frequency-tunable oscillator. Basic research for various applications—including imaging, spectroscopy, high-capacity wireless communication, and radar systems—of RTD oscillators has been carried out. Some recent results relating to these applications are discussed.

## 1. Introduction

The terahertz (THz) band, which has a frequency of about 0.1 to several THz, is expected to play key roles in various applications, such as imaging, chemical and biotechnological analyses, and communications [1,2,3]. Compact solid-state THz sources are important devices for these applications and various kinds of such sources have been studied, comprising both optical and electronic devices, as the THz band is located between millimeter and light waves.

**Figure 1:**
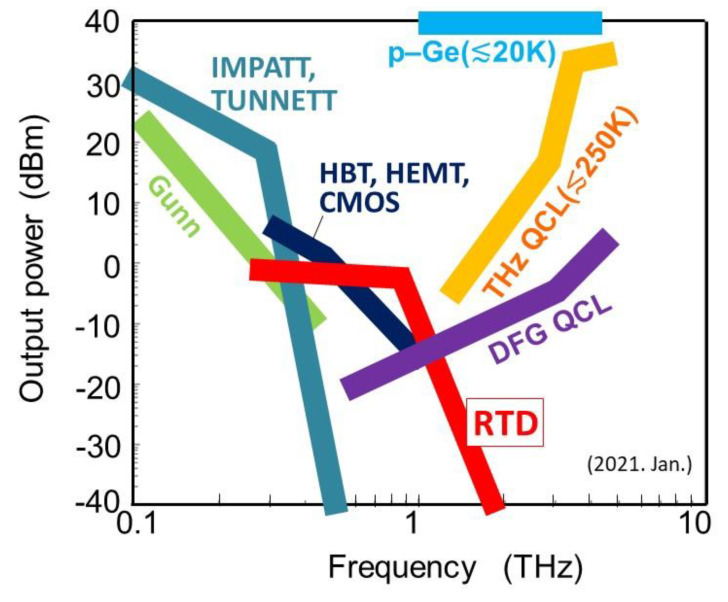
Current status of semiconductor on-chip terahertz (THz) sources. Output power as a function of generated frequency. Devices without temperature indication operate at room temperature.

Figure 1 shows the current status of the various semiconductor THz sources that directly generate THz waves from a dc power supply—note that sources that require other external microwave or light sources to generate THz waves (e.g., by multiplication or difference frequency) are not included in the figure. On the optical device side, p-germanium (p-Ge) lasers [4] and quantum cascade lasers (QCLs) have been studied [5,6,7,8,9]. Recently, room-temperature THz sources with difference frequency generation (DFG) using mid-infrared QCLs have been reported [10,11,12,13]. These devices are included in Figure 1, as all the light sources needed for these devices are integrated into one chip and the THz waves are generated by DC power supplied to the chip without external sources of other frequencies. On the electronic device side, impact ionization avalanche transit-time (IMPATT) diodes, tunneling transit-time (TUNNETT) diodes, Gunn diodes [14,15,16], and transistors such as heterojunction bipolar transistors (HBTs), high electron mobility transistors (HEMTs), and complementary metal-oxide-semiconductor (CMOS) transistors [17,18,19,20,21] have been studied as THz sources. Recently, the operating frequencies of transistors have increased remarkably. Other than semiconductors, THz emitters using intrinsic Josephson junctions in the layered high-temperature superconductor Bi_2_Sr_2_CaCu_2_O_8+δ_ have also been studied [22,23], which have the advantage of having a wide frequency tuning range.

Resonant-tunneling diodes (RTDs) are also promising candidates for room-temperature THz sources [24,25,26,27,28,29,30,31]. Currently, oscillation up to 1.98 THz has been obtained at room temperature [32,33], while structures for higher frequency and high output power are being studied [34,35]. Studies aiming toward several applications, such as imaging, spectroscopy, wireless communications, and radars, have recently begun [36,37,38,39,40,41,42,43,44,45,46,47]. In this paper, we review recent developments and applications of THz sources using RTD oscillators. Although RTDs can also be used as THz detectors [48,49,50,51], here, we only describe RTD THz sources, except for a brief introduction of the application of RTD THz detectors for wireless communications.

## 2. Structure, Oscillation Principle, and Oscillation Characteristics of RTD Oscillators

**Figure 2:**
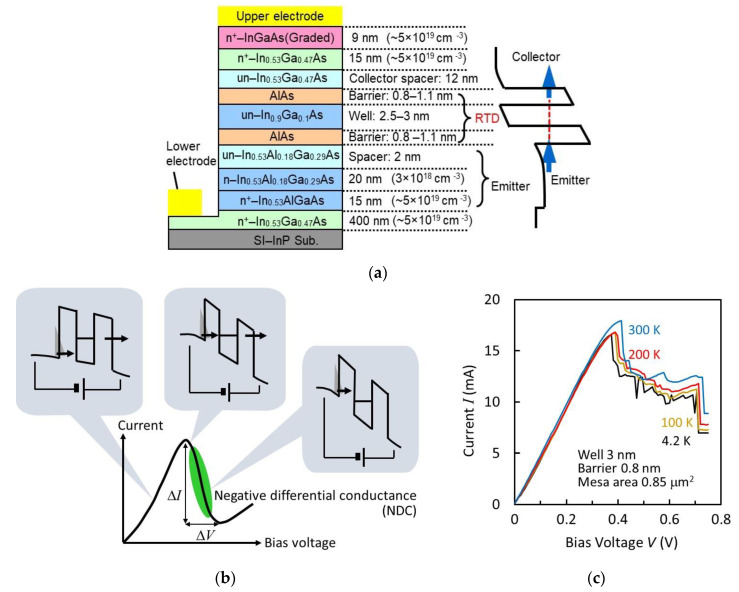
Layer structure and current–voltage characteristics of resonant-tunneling diodes (RTDs): (**a**) Layer structure of InGaAs/AlAs double-barrier RTD. Reprinted with permission from [56]. Copyright (2016) Springer Nature. (**b**) Schematic I–V curve and potential profile at various bias voltages, and (**c**) an example of measured I–V curves at various temperatures [52].

An RTD is made of heterostructures with ultrathin semiconductor multi-layers. The layer structure we use for a THz source is shown in Figure 2a. The main part is composed of an InGaAs quantum well and AlAs double barriers. An InGaAlAs emitter, an InGaAs collector spacer, and a high-doped InGaAs collector are constructed around the main part. These structures are epitaxially grown on a semi-insulating (SI) InP substrate. In DC operations, the conduction band edge of the emitter is lifted by bias voltage, as shown in Figure 2b. At the bias voltage where the conduction band edge of the emitter is aligned to or exceeds the resonance level of the quantum well, the current–voltage (I–V) curve indicates the negative differential conductance (NDC) region, in which the current decreases with increasing bias voltage. This region is used for the THz oscillation. In our RTD structure, a deep quantum well with indium-rich InGaAs and an emitter with InAlGaAs, having a high conduction band edge, are used to reduce the bias voltage required for NDC. Figure 2c shows an example of the measured I–V curves at various temperatures [52]. The NDC region can be seen to have unstable current fluctuation. This fluctuation occurs due to parasitic oscillations in the measurement circuits, composed of leading wires and power supply. The wires and power supply construct a resonance circuit for oscillation, which is described later. Relaxation oscillation [53] and current bi-stability which is caused by charge buildup and depletion in the quantum well [54,55] may also occur in this circuit.

The I–V curves change very little with temperature, probably as the carrier concentration at the conduction band edge of the emitter is insensitive to temperature, due to high Fermi energy, as well as because the AlAs barriers are high. The current density at the peak point is typically 10–30 mA/μm^2^, while the peak-to-valley current ratio (PVCR) is 2–4. The current density is large for narrow barriers and quantum wells, and strongly depends on the thicknesses of these layers.

As a material combination, we chose InGaAs/AlAs on an InP substrate, as high barriers and high current densities are possible in this system. For high output power, the large voltage width of the NDC region (ΔV in Figure 2b) is desirable, as discussed below. For this purpose, materials with high breakdown voltage may be advantageous. GaN-based material systems may be candidates, although high-frequency operations must be separately investigated. Some results of RTDs with such systems have been reported [57,58,59,60].

**Figure 3:**
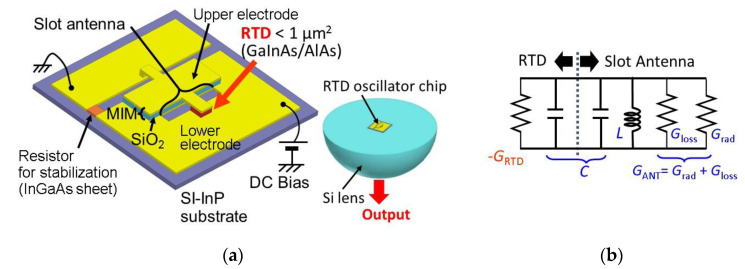
Structure and operating principle of RTD oscillators. Reprinted with permission from [56]. Copyright (2016) Springer Nature: (**a**) RTD oscillator integrated with slot antenna and entire structure including silicon hemispherical lens, and (**b**) equivalent circuit, including RTD and slot antenna. Parasitic elements in RTD are neglected for sake of simplicity.

The schematic structure of the fabricated RTD oscillator is shown in Figure 3a [56]. The RTD is placed near the center of one side of a slot antenna, which works as a resonator and a radiator, and the upper electrode of the RTD is connected to the other side of the slot through the capacitance formed by an MIM (metal–insulator–metal) structure. This MIM structure is used to isolate the bias lines to the upper and lower electrodes of the RTD. Outside of the slot antenna and RTD, a resistor for stabilization is connected in parallel with the RTD to suppress parasitic oscillations formed by the circuit, including the leading wires and power supply. By making the reciprocal of this resistor larger than the absolute value of the NDC of the RTD, the NDC is electrically hidden from the outside. As shown in the right-hand side of Figure 3a, the oscillator chip is mounted on a silicon hemispherical or hyper-hemispherical lens, in order to extract the output power, as most of the output is radiated from the slot antenna to the substrate side, due to the large dielectric constant of InP [61]. For a collimated output beam, a hyper-hemispherical lens is used. Structures without silicon lenses have also been reported [27,28,62,63,64].

Figure 3b shows the equivalent circuit of the oscillator in the THz frequency region, where $-G_{RTD}$ is the NDC of the RTD, $G_{ANT}$ is the conductance of the slot antenna, which is composed of the radiation conductance $G_{rad}$ and the conductance due to the Ohmic loss $G_{loss}$, and L and C are the inductance and capacitance of the RTD and slot antenna. As the capacitance of the RTD is much larger than that of the slot antenna, C is dominated by the RTD, while L is dominated by the slot antenna. In the device design, L and C are calculated for the antenna using three-dimensional (3D) electromagnetic simulation and the parallel-plate capacitances of the RTD are calculated for the constituent layers. The additional capacitance caused by the electron delay time is also considered in the RTD [65]. Parasitic elements [56,65] around the RTD are neglected in Figure 3b, for the sake of simplicity in the explanation of oscillation principle.

The condition required for oscillation is $G_{RTD}\geq G_{ANT}$ at the oscillation frequency $f_{OSC}=1/\left(2\pi \sqrt{LC}\right)$. As the oscillation frequency is determined by the total of *LC* formed by the antenna and RTD, the length of the antenna is usually much shorter than the half-wavelength of the oscillation frequency. For a fixed antenna structure, the oscillation frequency can be increased by reducing the capacitance of the RTD, which is mainly done by reducing the RTD mesa area. However, $G_{RTD}$ simultaneously decreases with the reduction of the RTD mesa area. Thus, the oscillation frequency reaches its upper limit ($G_{RTD}=G_{ANT}$) with the reduction of the RTD mesa area. In addition, due to the delay time of electrons in the RTD layers, $G_{RTD}$ per area also degrades with increasing frequency.

The above description of the oscillation principle is based on NDC in electrical circuits. As the frequency increases, the photon energy becomes non-negligible and a different explanation, including electron transitions, is needed (as in a laser). However, as the amplification of electromagnetic energy can be expressed by an equivalent circuit, the above electrical description can be used as an approximate one, by changing parameters such as NDC.

Considering the above conditions, the requirements for an RTD to obtain high oscillation frequency are high $G_{RTD}$ per area at high frequency and low capacitance per area. Small values of $G_{loss}$ and L are also required for the antenna. $G_{rad}$ in $G_{ANT}$ cannot be reduced, as the output power is determined by $G_{rad}$ (see below). Although the parasitic elements around the RTD also degrade $G_{RTD}$ with increasing frequency, the other effects mentioned above seem to be significant, so far, to increase the oscillation frequency [56,65,66]. At higher frequencies, the effects of the parasitic elements need to be considered in detail.

In order to increase $G_{RTD}$, the current density in the I–V curve is increased with thin barriers and the quantum well, as shown in Figure 2a. The capacitance per unit area is also reduced by inserting the collector spacer layer in Figure 2a. For the electron delay time in RTD layers, the degradation of $G_{RTD}$ with frequency is discussed using the approximate formula $G_{RTD}\left(\omega \right)\propto \cos\left[\omega \left(\tau_{RTD}+\tau_{dep}/2\right)\right]$, where $\tau_{RTD}$ and $\tau_{dep}$ are the residence time in the double barrier structure and the transit time in the collector spacer layer, respectively [56,65,66]. In the derivation of this formula, $\tau_{RTD}$ is phenomenologically introduced by assuming that electrons are affected only by the time delay $\tau_{RTD}$ at resonant tunneling [65]. A detailed analysis for a more exact treatment is a future subject, including, for example, the potential change due to electron accumulation in the well [67,68], photon-assisted tunneling [69,70,71], and so on, or more precise quantum-mechanical analyses [72,73,74]. In fact, the experimental result of the frequency dependence of $G_{RTD}$ [52] slightly deviated from the above formula, although more experimental data are needed.

In any case, it is clear that the delay time must be reduced for higher-frequency oscillation. We used thin barriers and a quantum well to reduce the delay time at resonant tunneling, in addition to high current density [75]. Furthermore, we optimized the thickness of the collector spacer to make $\tau_{dep}$ and the capacitance as small as possible at the same time. Using these methods, oscillation frequencies up to 1.42 THz have been obtained [76]. The length of the slot antenna was fixed at 20 μm, while the oscillation frequency was increased by reducing the RTD mesa area. The RTD mesa area was approximately 0.6 μm^2^ at 1 THz and 0.2 μm^2^ at 1.42 THz. The output power was approximately 20 and 1 μW at around 1 and at 1.42 THz respectively, and rapidly decreased as the RTD mesa area approached the upper limit of oscillation.

For the antenna, $G_{loss}$ can be reduced by reducing the conduction loss, which exists on the metal surface around the slot and on the bridge connecting the antenna to the RTD. The former was reduced by optimizing the combination of antenna length and RTD mesa area, through which oscillation up to 1.55 THz has been obtained [77]. The latter was also reduced by improving the structure of the bridge. Through the use of these methods, oscillation up to 1.92 THz has been obtained [78].

**Figure 4:**
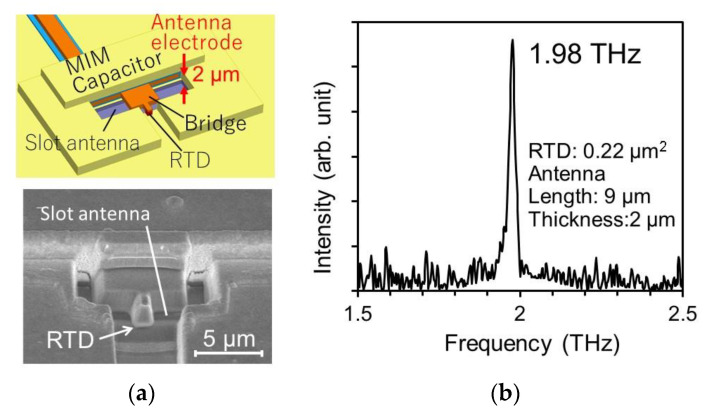
RTD oscillator with thick antenna electrode. Copyright (2017) IEEE. Reprinted with permission from [32]: (**a**) Structure of the oscillator, and (**b**) oscillation spectra.

In addition, by making the antenna electrode thicker, the area of the side wall of the slot increases and the conduction loss is further reduced. Combining all of the methods mentioned above, oscillation frequency up to 1.98 THz has been obtained [32], as shown in Figure 4. This is the highest frequency achieved by room-temperature electronic single oscillators, to date.

However, the decrease in $G_{loss}$ saturated with a further increase in thickness of the antenna electrode [33]. This was because the inductance $L_{slot}$ of the slot antenna also decreases with increasing antenna thickness, in addition to the decrease in the resistance R of the antenna electrode. As R is connected in series with $L_{slot}$ and the relation $R\ll \omega_{osc}L_{slot}$ holds at the angular frequency of oscillation $\omega_{osc}$, $G_{loss}$ can be approximately given by $R/{\left(\omega_{osc}L_{slot}\right)}^{2}$. $G_{loss}$ remarkably decreases with increasing antenna thickness, up to approximately 2 μm [33], due to the decrease in R and the weak dependence of $L_{slot}$ on antenna thickness. Above this thickness, however, $G_{loss}$ saturates with antenna thickness due to the decrease in $L_{slot}$. Thus, the upper limit of oscillation frequency saturates with the antenna thickness. Considering this result, a new structure other than the slot-integrated one must be proposed for higher-frequency oscillation, as shown in the next section.

The output power of an RTD oscillator is theoretically given by $P_{out}=\left(2/3\right)G_{rad}\left(G_{RTD}-G_{ANT}\right)/b$, where b is the coefficient of the non-linear term included in the NDC under oscillation [65]. $P_{out}$ changes with $G_{rad}$, and is maximized at $G_{rad}=G_{RTD}-G_{ANT}$, i.e., $G_{rad}=\left(G_{RTD}-G_{loss}\right)/2$. Using the third-order polynomial approximation of the I–V curve, $G_{RTD}$ and b can be expressed as [65] (3/2)(ΔI/ΔV) and $2\Delta I/\Delta V^{3}$ respectively, where ΔI and ΔV are the current and voltage widths in the NDC region of the I–V curve, as shown in Figure 2b. The maximum output power in the above condition is calculated as $P_{max}=\left(3/16\right)\Delta I\Delta V{\left(1-G_{loss}/G_{RTD}\right)}^{2}$. Thus, in order to increase the output power, $G_{rad}$ must be optimized, ΔI and ΔV must be increased, and $G_{loss}$ must be reduced. The oscillators integrated with slot antennas described above are not optimized for $G_{rad}$, and their typical output power is a few tens of μW. The $G_{rad}$ of the slot antenna can be designed and optimized through the offset structure, in which the position of the RTD is shifted from the center of the slot and an output power of a few hundred μW has been obtained [79,80]. ΔI can be increased by increasing the RTD mesa area; however, the oscillation frequency decreases, due to an increase in capacitance. A structure with a large ΔI and small $G_{loss}$ that can maintain the oscillation frequency is shown in the next section. The increase of ΔV is a future subject. A possible method may be through the appropriate design of RTD layers (e.g., an increase in thickness of the collector spacer layer), although the upper limit of oscillation frequency must be discussed simultaneously.

**Figure 5:**
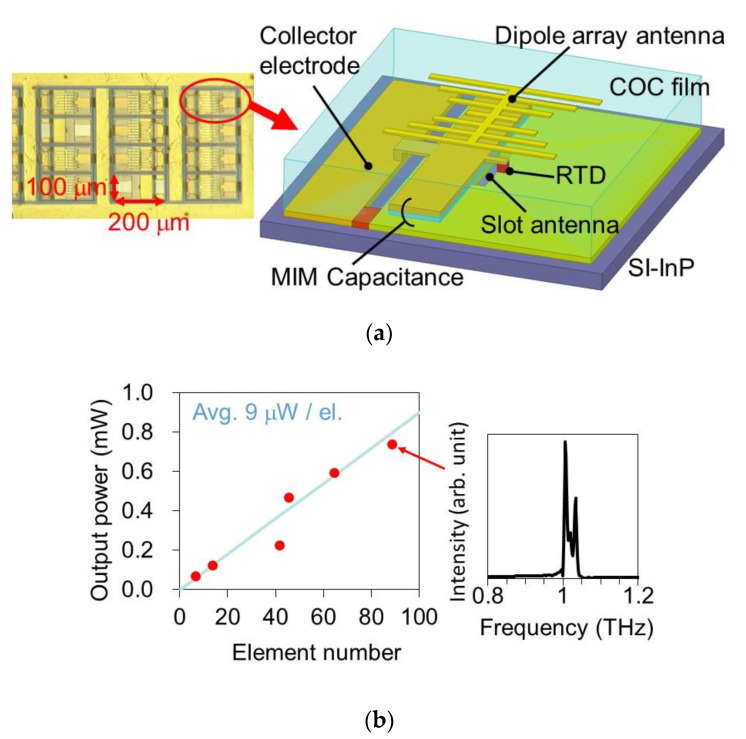
Large-scale array of RTD oscillators. Reprinted from Reference [63] with the permission of AIP Publishing: (**a**) Structure of the device—an array element is composed of an RTD with a slot antenna (resonator) covered by COC (cyclic olefin copolymer) film and a dipole array antenna (radiator), and (**b**) output power as a function of element number and oscillation spectrum for 89-element array.

Power combining through array configuration is also useful for obtaining high output power. An oscillator with a two-element array of the offset slot antennas has exhibited an output power of 0.6 mW at 620 GHz [80]. In this array, single-frequency oscillation was observed due to mutual locking between the coupled elements, which implies coherent power combining. In a large-scale array without intentional coupling between the elements, 0.73 mW has been obtained at 1 THz for 89 elements, as shown in Figure 5 [63]. In this device, any intentional coupling structure for stable synchronization was not introduced. However, the elements appeared to be weakly coupled with each other through random reflections and feedback of the output power radiated into the substrate or the dielectric film (COC film in Figure 5). As the elements were not perfectly synchronized, due to weak coupling, multiple peaks were observed in the oscillation spectrum. This behavior is suitable for applications such as imaging in which the interference fringe is a problem in coherent sources.

For stable synchronization and coherent power combining, strong coupling between array elements is required; furthermore, as the number of elements increases, stronger coupling is required [81]. As coupling through the circuits on the element plane seems to be limited, another method may be needed, such as putting the entire array into a resonator for strong coupling.

The measurement of the temperature dependence of oscillation characteristics has also been reported [52]. The oscillation frequency was almost constant with temperature, while the output power drastically increased with decreasing temperature between 10 and 300 K. As NDC is insensitive to temperature, as can be seen from Figure 2c, the change in output power was attributed to the change in Ohmic loss of the antenna electrode with temperature. In the narrow temperature range of 300–350 K, the change in the measured output power was small.

## 3. Novel Oscillator Structures for High Frequency, High Output Power, and Easy Fabrication

**Figure 6:**
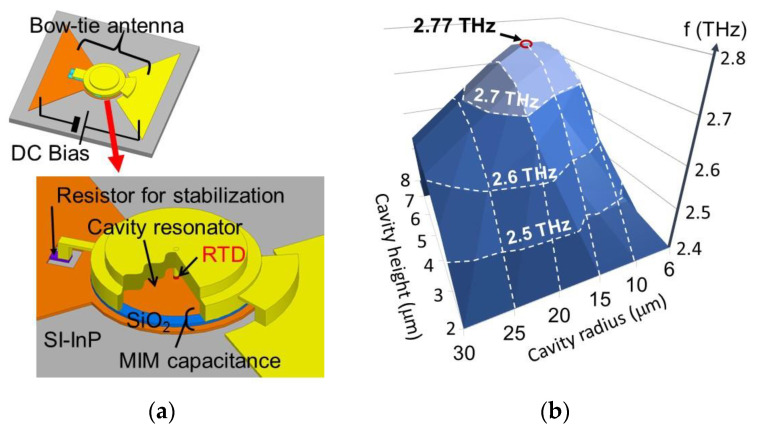
RTD oscillator integrated with cylindrical cavity and bow-tie antenna for high-frequency oscillation [33,34]: (**a**) Oscillator structure, and (**b**) calculation of oscillation frequency as a function of cavity height and radius. Copyright (2020) The Japan Society of Applied Physics [34].

As the upper limit of oscillation frequency of slot-integrated oscillators was found to saturate with increasing thickness of antenna electrode, as mentioned in the previous section, a novel oscillator structure with a cylindrical-cavity resonator and bow-tie antenna has been proposed for higher frequency oscillation, as shown in Figure 6a [33]. In this structure, the oscillation frequency is determined by the resonance frequency of the resonator composed of the cavity and the RTD, and the output is radiated from the bow-tie antenna, which is connected to the cavity by the MIM layers. As the surface area of the cavity is large, the conduction loss is small and high-frequency oscillation is expected. A theoretical calculation [34] has shown that oscillation up to approximately 2.8 THz is possible by optimizing the radius and height of the cylindrical cavity, as shown in Figure 6b. In a preliminary experiment, 1.78 THz oscillation has been obtained for a non-optimized structure with parasitic capacitance [33]. The theoretical output power is approximately 0.5–1 μW around 2.5 THz, at present [82]. Higher frequency and higher output power can be expected with further structure optimization, including other parameters. For example, the output power can be increased with the capacitance of MIM structure connecting the cavity and antenna, as the antenna conductance (viewed from RTD) is altered by this capacitance and can be optimized, as mentioned in the previous section. An array configuration is also useful for high output power. Even with a small output power in a single oscillator, there may be suitable applications, such as microchips for spectroscopy, as explained later. Fabrication of an oscillator using a cylindrical cavity is currently in progress.

**Figure 7:**
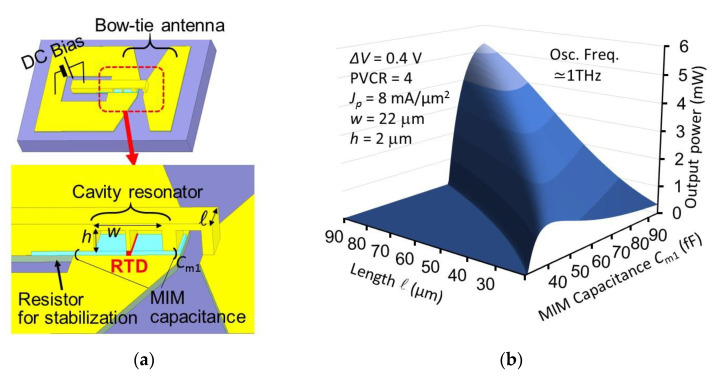
RTD oscillator integrated with rectangular cavity and bow-tie antenna for high output power [35,83]: (**a**) Oscillator structure, and (**b**) calculation of output power as a function of cavity length and capacitance at the metal-insulator-metal (MIM) structure connecting bow-tie and cavity.

For high output power, an oscillator integrated with rectangular cavity has been proposed [35,83], as shown in Figure 7a. In this structure, the RTD has a long strip shape, and the cavity is regarded as a small inductance connected to the RTD. Due to the low inductance of the cavity, an RTD with large capacitance and large mesa area can be used for oscillation at relatively high frequency. Thus, the current width ΔI of the NDC region, to which the maximum output power is directly related, becomes large due to the large-area RTD. In addition, the large surface area of the cavity reduces the conduction loss, which is also effective for high output power. Figure 7b shows the results of a theoretical calculation of the output power as a function of cavity length and capacitance at the MIM structure which connects the bow-tie antenna to the cavity for output radiation [83]. The conductance of the antenna, as viewed from the RTD, is controlled by the MIM capacitance, where the optimum condition for maximum output power mentioned in the previous section can be achieved. At the optimum MIM capacitance, the output power monotonously increases with cavity length, due to the increase in ΔI. Although heating due to the large current should carefully be considered in an actual device, an output power of 3–5 mW at 1 THz is theoretically expected with a cavity length of 50–70 μm, even for a single oscillator. Figure 7b shows only the output power from the bow-tie antenna, and does not include radiation from the open surfaces of the rectangular cavity. The radiation from these two open surfaces of the cavity cancel each other out in the direction directly above the cavity, as they are in an anti-phase relationship. The fabrication of this structure is also currently in progress. The oscillation frequency cannot be as high as that of the cylindrical-cavity structure mentioned above, as an extremely narrow strip of the RTD mesa is required for high frequency, but this structure is suitable for high output power with a single element.

**Figure 8:**
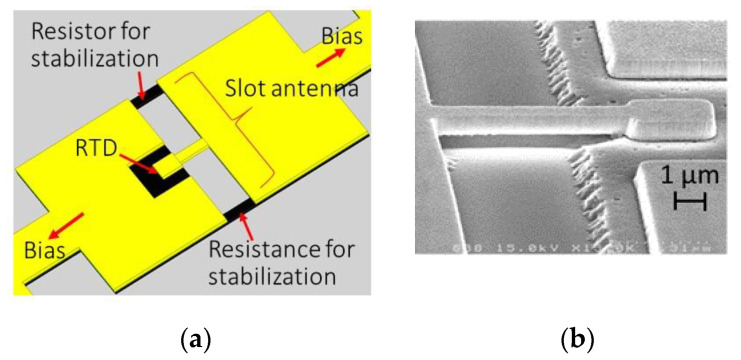
Simplified structure of RTD oscillator integrated with slot antenna. RTD oscillator (**a**) simplified steucture of RTD oscilator integrated with slot antenna; (**b**) SEM image of the fabricated airbridge. Reprinted with permission from Reference [84]. Copyright (2020) Springer Nature.

Figure 8 shows a simplified structure of an RTD oscillator integrated with the slot antenna proposed for easy fabrication [84]. As this structure does not have the MIM layers needed for the previous oscillator in Figure 3a, the requirements of the lithography processes are reduced, such that the fabrication is easier and can be completed in a short period of time. In this structure, the resistor for stabilization made by the InGaAs layer is located inside the slot antenna, in contrast to the previous structure shown in Figure 3a. Similar to the resistance of the antenna electrode discussed in the previous section, which is connected in series with the inductance $L_{slot}$ of the slot antenna, the conductance caused by the resistor for stabilization, as viewed from the RTD, is approximately given by $R_{s}/{\left(\omega_{osc}L_{slot}\right)}^{2},$ where $R_{s}$ is the value of the resistor for stabilization. The conductance, as viewed from the RTD, is much smaller than the real conductance, $1/R_{s}$. Thus, it is possible to design $R_{s}$ to satisfy $R_{s}/{\left(\omega_{osc}L_{slot}\right)}^{2}\ll G_{RTD}<1/R_{s\;}$, such that the resistor for stabilization can suppress the parasitic oscillations at low frequency without large loss, in terms of the oscillation frequency.

In a preliminary experiment, oscillation up to 740 GHz has been obtained for a 30 μm-long antenna, with output power of approximately 10 and 1 μW at around 600 and 740 GHz, respectively [84]. Higher oscillation frequencies are expected with shorter antennas. These characteristics were comparable to the previous structure, although the upper limit of oscillation frequency may be slightly lower than that of the previous structure, due to the effect of the resistor for stabilization. As this structure does not require complicated three-dimensional integration, it can easily be extended to various planar structures, such as high-density arrays, metamaterials including RTDs, and so on.

## 4. Frequency Tuning, Spectral Narrowing, and Different Polarizations

**Figure 9:**
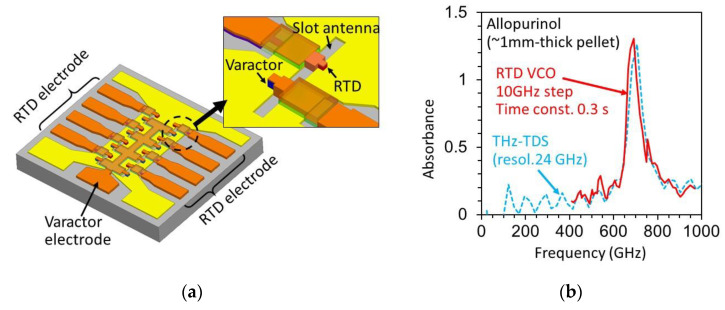
Frequency-tunable RTD oscillator and application to absorbance measurement: (**a**) Array of frequency-tunable oscillators integrated with varactor diodes, and (**b**) absorbance measured by RTD oscillators together with the result of THz time-domain spectroscopy (TDS) measurement. Copyright (2017) The Japan Society of Applied Physics 47.

Frequency and spectrum control are important for various applications, such as spectroscopy. In the RTD oscillators mentioned above, the oscillation frequency was almost fixed at the frequency determined by the structure, except for small changes (1–5%) with bias voltage [85]. As a voltage-controlled frequency-tunable oscillator (VCO), an oscillator integrated with a varactor diode in the slot antenna has been reported [86,87,88]. The varactor diode is made of a pn junction of InGaAs, where the oscillation frequency can be electrically varied by changing its capacitance and resistance using reverse [86] or forward [87] bias voltage. By optimizing the areas of the varactor diode and RTD, a frequency change of about 100 GHz was obtained. Moreover, using an array configuration of frequency-tunable oscillators with different frequency ranges, a wide frequency change has been reported [88]. Figure 9 shows an array of frequency-tunable RTD oscillators and its application to absorbance measurement [47]. Using the frequency change of 410–970 GHz in the RTD oscillators, the absorbance of allopurinol was measured. The results agreed with that of conventional THz time-domain spectroscopy (TDS), within the resolutions of these two measurements.

By integration of the frequency-tunable RTD oscillator with a detector through a transmission line and a space for a droplet of specimen, a microchip which can be used for spectroscopy is expected. Fast measurements may be possible, even with low output power of the oscillator, due to the short distance between the source and detector. Neglecting the transmission loss between the source and detector, the output power required for the oscillator is estimated to be $P=\mathrm{SNR}\cdot \mathrm{NEP}/\sqrt{2\pi T}\;∼\;$0.6 μW, with the signal-to-noise power ratio (SNR) at the detector (dynamic range) of 30 dB, the time constant *T* of 1 ms, and the noise equivalent power (NEP) of 50 pW/Hz^1/2^, which is typical for Schottky barrier diodes (SBDs).

For the spectral line width, a full width at the half maximum (FWHM) of about 10 MHz has been reported in an RTD oscillator with output power of about 1 μW [89]. The line width is determined by the phase noise caused by the shot noise of the RTD, which is calculated as $\Delta f=\left(\pi /4\right)\left(p_{n}\Delta f_{r}^{2}/P_{out}\right)$ in FWHM [90], where $p_{n}$ is the noise power density per frequency included in the output power $P_{out}$, and $\Delta f_{r}$ is the line width in FWHM of the resonator system with the RTD capacitance and the slot antenna and without NDC, which is expressed as $\Delta f_{r}=f_{OSC}/Q$, using the oscillation frequency $f_{OSC}$ and the Q factor of this resonator system. From this equation, the line width can be reduced by an increase in the product $P_{out}Q^{2}$. Spectral narrowing with high Q factor by an RTD efficiently coupled to a photonic-crystal cavity has been reported [91].

**Figure 10:**
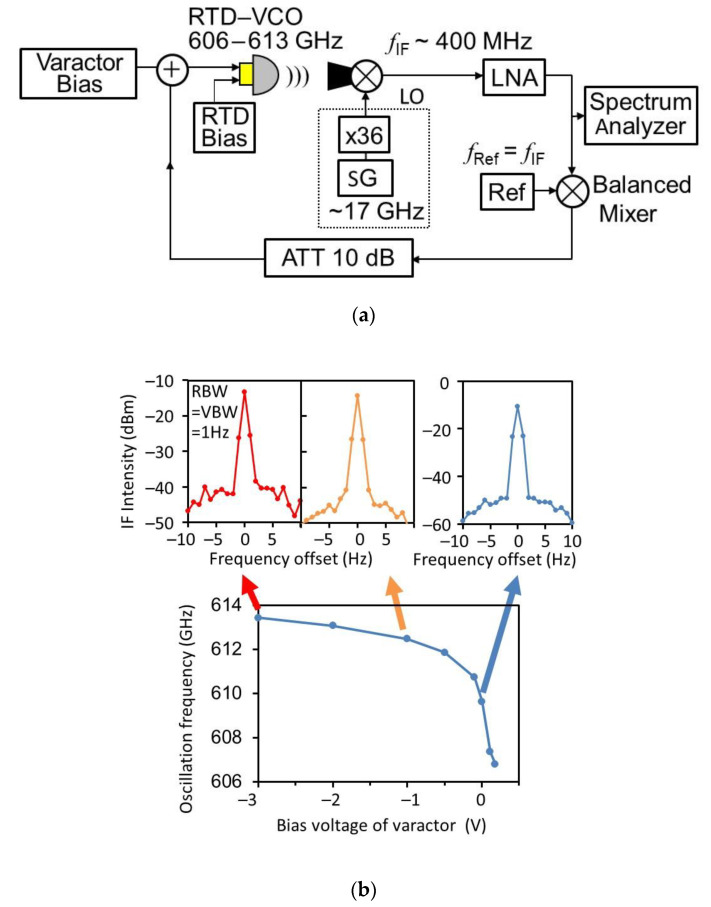
Phase-locked loop (PLL) system and spectral narrowing of frequency-tunable RTD oscillator. Copyright (2018) The IEICE of Japan [93]: (**a**) Block diagram of the PLL system—LNA: low-noise amplifier, ATT: attenuator, SG: signal generator, and (**b**) oscillation spectra under the PLL at various oscillation frequencies—RBW: resolution band width, VBW: video band width.

Spectral narrowing by a phase-locked loop (PLL) has also been reported [92,93]. Figure 10a shows a PLL system for a frequency-tunable RTD oscillator. By converting the output of the RTD oscillator to a low-frequency signal by heterodyne detection, extracting the phase noise by mixing the converted signal with the reference signal, and feeding it back to the varactor diode in the oscillator, a line width as narrow as 1 Hz has been achieved, as shown in Figure 10b).

Reduction in the spectral line width of an RTD oscillator by external sub-harmonic injection locking has also been reported [94]. External injection locking is also useful for phase control of the output. Under injection locking, the oscillation frequency of the RTD oscillator is locked to that of the external injection, even if the free-running frequency of the RTD oscillator is slightly different from that of the injection. An operation changing the frequency under the free-running condition (i.e., changing the bias voltage of the varactor diode or RTD) results in a phase change of the output under the locked condition. Utilizing this property for the injection locking of an uncoupled array of RTD oscillators, a phased array can be constructed, which has the beam steering function of the output which is useful for various THz applications. Phase changing of RTD oscillators by fundamental and sub-harmonic injection locking has been theoretically analyzed [95].

**Figure 11:**
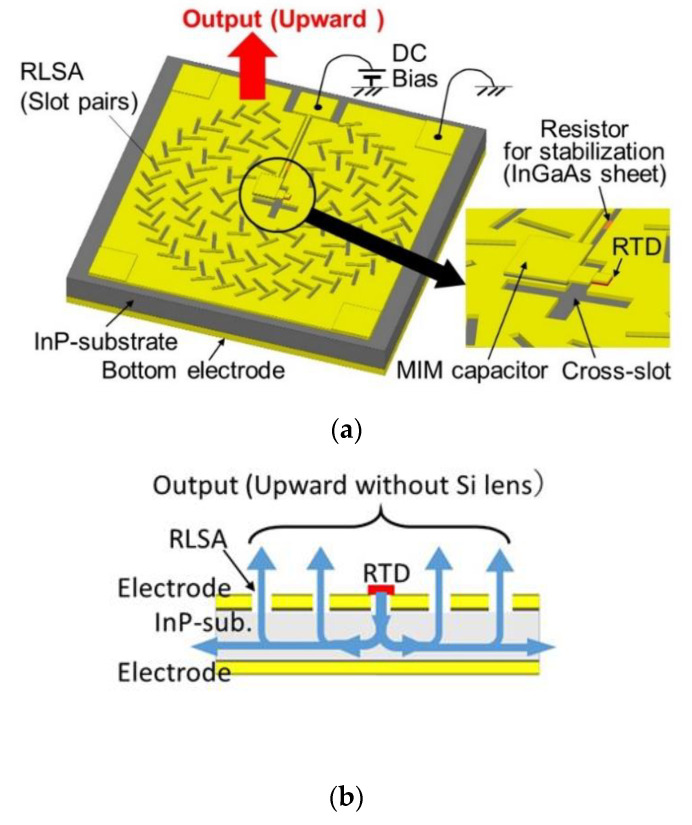

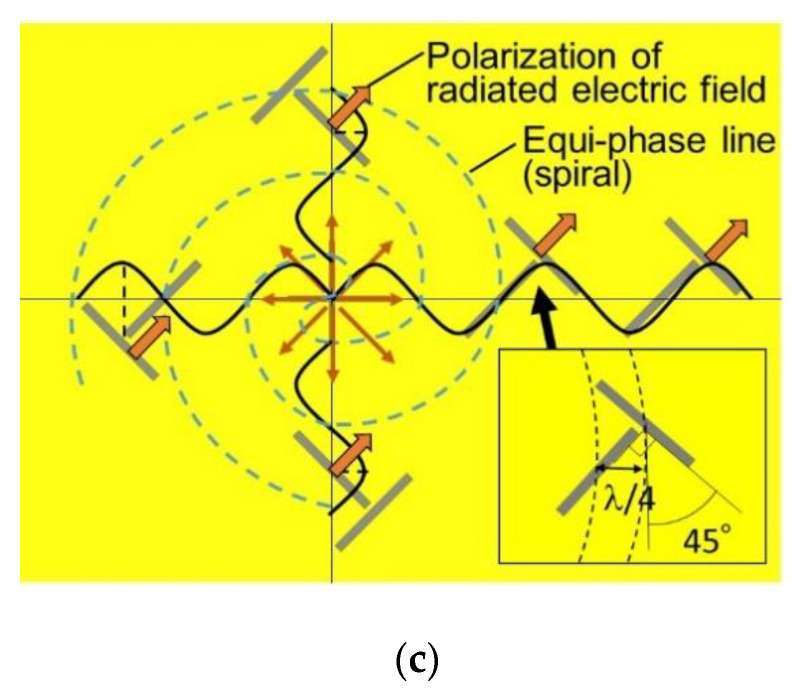
RTD oscillator integrated with radial line slot array (RLSA) for radiation of circularly polarized wave [64]: (**a**) Top view, (**b**) cross-sectional view, together with propagation of output from RTD, and (**c**) top view, together with phase distribution of the propagating wave in the substrate.

In the above oscillators, the output is linearly polarized. An RTD oscillator integrated with a radial line slot array (RLSA), which radiates a circularly polarized wave, has also been reported [64]. The structure of this oscillator is shown in Figure 11. The RTD is located at one of the cross-slot antennas, as shown in Figure 11a, and radiates the output wave into the substrate side. The radiated wave propagates along the substrate and is emitted from the elements of the RLSA around the RTD in the upward direction, as shown in Figure 11b. Each element of the RLSA radiates the circularly polarized wave. These elements are arranged concentrically and radiate output waves in phase, as shown in Figure 11c, resulting in high directivity without a lens. An axial ratio of 2.2 dB for the circularly polarized output and the directivity of 15 dBi were obtained at 500 GHz. The circularly polarized output is useful in preventing the influence of external feedback that causes fluctuations of the oscillation characteristics of the RTD [96]. By changing the arrangement of the elements from concentric to spiral, the radiation of a vortex wave, which can be used for multiplex wireless communications with different rotation numbers, has also been obtained [97].

## 5. Applications

Basic research into various applications of RTD oscillators has begun, including for imaging [36], sensors [37], linear encoders [38], communication [39,40,41,42,50], and radars [43,44,45,46], in addition to spectroscopy [47] shown in the previous section. It is a future task to develop various applications of RTD oscillators. Here, we briefly introduce recent applications, especially with respect to communication and radar.

**Figure 12:**
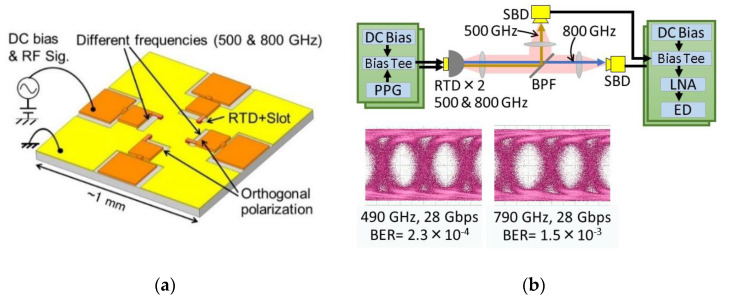
RTD oscillators with different frequencies and polarizations integrated into one chip for wireless transmission with frequency and polarization multiplexing. Copyright (2017) IEEE. Reprinted with permission from [40]: (**a**) Schematic structure of the integrated chip, and (**b**) diagram of the transmission with frequency multiplexing and the resulting eye diagrams—PPG: pulse pattern generator, ED: error detector, BER: bit-error rate, BPF: band-pass filter.

As the output of RTD oscillators can easily be intensity-modulated by direct modulation (i.e., superposition of a signal on the bias voltage), simple high-capacity THz wireless communications are possible. The upper limit of the direct modulation frequency of 30 GHz has been reported [98], which is limited by the capacitance of the external circuit to impose the modulation signal onto the RTD. Simple on-off keying wireless data transmissions have been reported with a data rate of 44 Gbps and an error rate of 5 × 10^−4^ below the forward error correction (FEC) limit, and 25 Gbps without error at 650 GHz [39]. Preliminary experiments on transmissions with frequency and polarization multiplexing using RTD oscillators have also been reported [40]. By integrating oscillators having two orthogonal polarizations and two frequencies of 500 and 800 GHz on the same substrate, transmission of 2 × 28 Gbps was obtained, with error below FEC limit in both the frequency and polarization multiplexing. These are also simple on–off keying data transmissions. Figure 12 shows the oscillator chip for frequency and polarization multiplexing, the diagram of the frequency multiplexing, and the transmission result. By improving the external circuit around the RTD for the modulation signal, higher data rates are expected. A transmission experiment using an RTD oscillator with a circularly polarized wave has also been reported [64]. Although the data rate was still low (1 Gbps), it was shown that the error rate was insensitive to oscillator rotation.

Wireless transmission using RTDs as detectors has also been reported [49,50]. The RTD is expected to have a high sensitivity in THz detection, due to the strong non-linearity in the I–V curve, which is the same principle as the detection in SBD (although bias voltage must be applied to use the strong non-linearity in RTD). Other than the detection using such non-linearity, a self-homodyne THz detection mode has recently been reported [50,51]. In this mode, the THz signal is detected by an RTD oscillator which is oscillating near the frequency of the irradiated signal. Through this irradiation, the RTD oscillator is injection-locked and a signal with the homodyne detection is obtained. Through this operation, a low value of NEP (7.7 pW/Hz^1/2^) has been obtained [51].

The application of RTD oscillators to THz radar has also been studied [43,44,45,46]. The THz radar has the advantage that it can be used in environments with poor visibility, due to the transparency of THz waves. 3D transparent imaging is also possible by combining THz radar and two-dimensional (2D) imaging systems.

**Figure 13:**
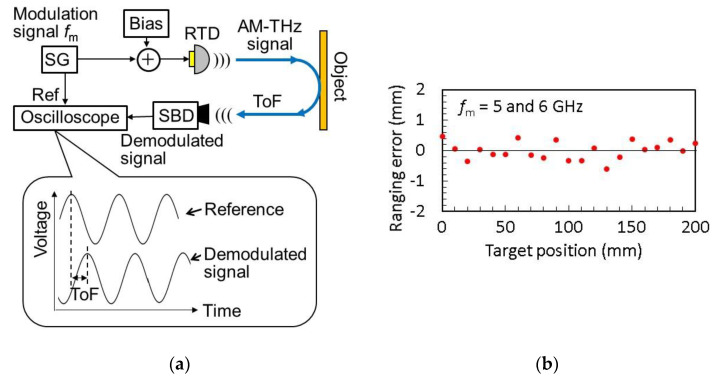
THz radar system using RTD oscillator [43,44]: (**a**) Simplified schematic diagram of THz radar system using RTD oscillator with the amplitude-modulated continuous wave (AMCW)—SG: signal generator, and (**b**) error evaluated for the distance measurement with the AMCW using two modulation frequencies.

Figure 13 shows a simplified schematic diagram of a system and measurement results of THz radar using an RTD oscillator [43,44]. This system uses the amplitude-modulated continuous wave (AMCW) method. In Figure 13a, the output of the RTD is amplitude-modulated by superimposing a sinusoidal signal on the bias voltage, which is then irradiated onto an object. The reflected wave from the object is received and demodulated by SBD. The time of flight (ToF) of the THz wave from RTD to SBD is determined by the phase difference between the demodulated and reference signals, from which the distance to the object is obtained.

In this method, when the phase difference between the demodulated and reference signals exceeds 2π, the number of periods included in the phase difference cannot be extracted. To solve this problem, two slightly different frequencies are used for modulation [43]. The phase difference is measured for each frequency, in order to extract the number of the periods included in the phase difference. By utilizing the fact that the period number must be an integer, the error in this number caused by noise is totally removed and high accuracy in the phase evaluation can be obtained.

Furthermore, the oscilloscope in Figure 13a can be replaced with an In-phase/Quadrature (IQ) demodulation system, in order to obtain an accurate phase difference [44]. In the IQ demodulation, the demodulated signal from the SBD is separately mixed with the reference signal and its 90 degree-shifted signal, and two orthogonal components of the mixing output are obtained. The phase difference is calculated from the arctangent of the amplitude ratio of these two components. By introducing the above improvements, distance measurement with an error (standard deviation) of 0.063 mm has been achieved for the carrier frequency of 520 GHz, as shown in Figure 13b [44].

The system described above does not utilize the phase difference of the THz wave itself but, instead, the phase difference of the subcarriers superimposed on the THz wave of the RTD output. The features of THz waves can be used as the carrier. This method is very useful for RTD oscillators in which oscillation characteristics, such as frequency, are easily affected by the external feedback [96].

The subcarrier modulation method can be extended to other radar systems, such as the frequency-modulated continuous wave (FMCW) radar. A subcarrier FMCW radar using an RTD oscillator and a preliminary experiment for the distance measurement of two targets have been reported [45].

As another extension of subcarrier modulation, a method for measuring the distances of multiple targets has been proposed [46], the principle of which is similar to that of THz optical coherent tomography (OCT) [99]. By changing the modulation frequency (subcarrier frequency) $f_{m}$ in Figure 13a, the demodulated signal is obtained as a function of $f_{m}$. Then, the demodulated signal is decomposed to two orthogonal components by IQ demodulation. For example, for a single target, as shown in Figure 13a, the demodulated signal at the SBD is written as $V\cos\left[2\pi f_{m}\left(t-\tau \right)\right]$, where V is the amplitude of the demodulated signal reflected from the object and τ is the time delay of the demodulation signal to the reference signal, including propagation times in the space and cables. This signal is decomposed to two components, $V_{I}\cos\left(2\pi f_{m}t\right)+V_{Q}\sin\left(2\pi f_{m}t\right)$, where $V_{I}\left(f_{m}\right)=V\cos\left(2\pi f_{m}\tau \right)$ and $V_{Q}\left(f_{m}\right)=V\sin\left(2\pi f_{m}\tau \right)$. $V_{I}\left(f_{m}\right)$ and $V_{Q}\left(f_{m}\right)$ are extracted by the IQ demodulation as a function of $f_{m}$. By calculating the inverse Fourier transform of the complex function $V_{I}\left(f_{m}\right)-iV_{Q}\left(f_{m}\right)$, the distribution of the target positions can be obtained. For the above single-target case, $V_{I}\left(f_{m}\right)-iV_{Q}\left(f_{m}\right)=Ve^{-i2\pi f_{m}\tau }$ and the inverse Fourier transform gives Vδ(t−τ), assuming that the dependence of V on $f_{m}$ is weak. Thus, τ can be extracted and the position of the target found. For multiple targets, a superposition of this form with different values of τ is obtained, where the distribution of the target position is found. As the bandwidth of $f_{m}$ is finite in an actual measurement, the result of the inverse Fourier transform for a single target is not a δ-function but a pulse having a finite width approximately given by $1/\left(f_{max}-f_{min}\right)$, where $f_{max}$ and $f_{min}$ are the maximum and minimum values of $f_{m}$, respectively. This pulse width gives the resolution (in τ).

In a preliminary proof-of-concept experiment using this method, the distances of two targets were measured in the range of 20–200 mm, with an error (standard deviation) of approximately 0.6–2.5 mm for $f_{min}$ and $f_{max}$ of 3 and 18 GHz, respectively [46]. The error and resolution can be improved by an increase of modulation bandwidth and the use of RTD oscillators with high output power for high signal-to-noise ratio.

## 6. Conclusions

Recent developments and some applications of THz sources using RTD oscillators were described. A major feature of these sources is their compactness. Studies focused on high frequency, high output power, and various functionalities were discussed. An important issue to address in the future is obtaining high output power (at least more than 1 mW) with high efficiency at high frequency. In addition, advanced functions which are required for various applications, such as beam steering (which is important for communication and radar systems), must be investigated. On the other hand, there are some basic characteristics that are not yet fully understood, such as the THz response of electrons in RTD. We hope that future studies will make significant progress in this field, and that the range of various applications will be expanded.
